# Disrupted Topological Organization in Whole-Brain Functional Networks of Heroin-Dependent Individuals: A Resting-State fMRI Study

**DOI:** 10.1371/journal.pone.0082715

**Published:** 2013-12-17

**Authors:** Guihua Jiang, Xue Wen, Yingwei Qiu, Ruibin Zhang, Junjing Wang, Meng Li, Xiaofen Ma, Junzhang Tian, Ruiwang Huang

**Affiliations:** 1 Department of Medical Imaging, Guangdong No. 2 Provincial People's Hospital, Guangzhou, P. R. China; 2 Center for the Study of Applied Psychology, Key Laboratory of Mental Health and Cognitive Science of Guangdong Province, School of Psychology, South China Normal University, Guangzhou, P. R. China; University of Modena and Reggio Emilia, Italy

## Abstract

Neuroimaging studies have shown that heroin addiction is related to abnormalities in widespread local regions and in the functional connectivity of the brain. However, little is known about whether heroin addiction changes the topological organization of whole-brain functional networks. Seventeen heroin-dependent individuals (HDIs) and 15 age-, gender-matched normal controls (NCs) were enrolled, and the resting-state functional magnetic resonance images (RS-fMRI) were acquired from these subjects. We constructed the brain functional networks of HDIs and NCs, and compared the between-group differences in network topological properties using graph theory method. We found that the HDIs showed decreases in the normalized clustering coefficient and in small-worldness compared to the NCs. Furthermore, the HDIs exhibited significantly decreased nodal centralities primarily in regions of cognitive control network, including the bilateral middle cingulate gyrus, left middle frontal gyrus, and right precuneus, but significantly increased nodal centralities primarily in the left hippocampus. The between-group differences in nodal centralities were not corrected by multiple comparisons suggesting these should be considered as an exploratory analysis. Moreover, nodal centralities in the left hippocampus were positively correlated with the duration of heroin addiction. Overall, our results indicated that disruptions occur in the whole-brain functional networks of HDIs, findings which may be helpful in further understanding the mechanisms underlying heroin addiction.

## Introduction

Drug addiction, a major social problem, appears to be a chronic brain disease that involves complex interactions between biological and environmental variables and is characterized by a compulsive drive to take drugs despite serious negative consequences [1]. Heroin users are a major proportion of drug addicts, especially in China [2]. Since the development of neuroimaging technologies, many studies have been concerned with the mechanisms underlying drug addiction [3], [4], [5].

Resting-state functional magnetic resonance imaging (RS-fMRI), a non-invasive imaging technique, has been widely used to explore the intrinsic functional organization of the human brain [6], [7], [8]. Several studies that used this technique investigated heroin-related changes in spontaneous brain activity [9], [10], [11], [12], [13] and suggested that heroin addiction is related to widespread functional abnormalities in many brain regions. These regions include the amygdala [13], anterior cingulate cortex (ACC) [10], hippocampus [13], insula [13], lingual gyrus [9], orbitofrontal cortex (OFC) [9], and temporal cortex [10]. In addition, functional connectivity alterations have been found in heroin-dependent individuals (HDIs). Ma et al. [12] indicated that heroin users showed increases in functional connectivity between the nucleus accumbens and the ventral/rostral ACC and decreases in connectivity between the prefrontal cortex and the OFC as well as between the prefrontal cortex and the ACC. Liu et al. [11] detected abnormal connectivity between the prefrontal cortex, ACC, ventral striatum, insula, amygdala and hippocampus in heroin users. However, to date no study has considered heroin-related whole-brain functional networks during the resting-state.

Graph theory analysis provides a powerful tool for characterizing topological organization, including identifying global and nodal properties in whole brain functional networks. It has been applied to the study of normal brains [14], [15] and of various brain-related diseases, such as Alzheimer's disease [16], epilepsy [17], [18], depression [19], and schizophrenia [20], [21]. Although two previous studies [11], [22] explored brain functional networks in heroin addiction patients using graph theory analysis, these studies focused on regional functional connectivity [11] or on four specific circuits (control, reward, motivation/drive and memory) [22]. However, what heroin-related alterations occur in the whole-brain functional networks remains unknown. Because previous studies [9], [10], [11], [12], [13] have indicated that the brain connectivity alterations in HDIs are widespread, in this research we attempted to analyze the topological properties of whole-brain functional networks in HDIs based on graph theory.

In this study, we constructed brain functional networks with RS-fMRI data for a group of HDIs and a group of controls, and compared the topological organization of their brain networks using graph theory. In addition, considering the duration of heroin addiction as a vital clinical variable in understanding the effects of heroin on functional abnormalities [9], [23], [24], we analyzed the correlations between the altered network parameters and the duration of heroin addiction.

## Materials and Methods

### Subjects

We recruited seventeen heroin-dependent individuals (HDIs: 15 M/2 F, aged 26–50 years, mean ± *SD* = 36.29±6.86 years, right-handed) from the Addiction Medicine Division of Guangdong No. 2 Provincial People's Hospital. The HDIs were screened using the Structured Clinical Interview (SCID-IV) for the Statistical Manual of Mental Disorders, Fourth Edition (DSM-IV), to confirm the diagnosis of heroin dependence. Urine tests with a positive finding for heroin use were acquired before enrolling in the treatment program. None of the HDIs had used any other types of drugs according to a laboratory report and an interview conducted by a clinical psychologist (30 years of clinical experience). They were hospitalized for 6–7 days before RS-fMRI scanning took place, none of the HDIs used heroin, as confirmed by the medical personnel responsible for their care. All of HDIs except two were under daily methadone maintenance treatment at the time of study. In addition, we recruited fifteen age- and gender-matched normal controls (NCs: 12 M/3 F, aged 20–46 years, mean ± *SD* = 31.27±8.10 years, right-handed) as the normal controls. Table 1 lists the demographic details of all the volunteers in this study. The detailed clinical descriptions for each of HDIs are listed in Table S1.

**Table 1:** Demographic information for the heroin-dependent individuals (HDIs) and the normal controls (NCs) in the present study.

Characteristics	HDIs (*n* = 17)	NCs (*n* = 15)	*p*-value
Female/Male	2/15	3/12	0.645^a^
Age (years)	36.29±6.86	31.27±8.10	0.067^b^
Range (years)	26–50	20–46	
Education (years) Range (years)	10.24±3.21 2–15	11.07±4.11 5–17	0.526^b^
Head motion			
Translation (mm)	0.126±0.051	0.106±0.045	0.251^b^
Rotation (mm)	0.149±0.120	0.126±0.063	0.512^b^
Nicotine (Median, No. cigarette/day)	20 (0–40)	20 (0–40)	0.116^b^
Heroin use (years) Range (years)	9.21±5.28 1–19	N/A	
Heroin dosage (g/day) Range (g/day)	0.80±0.54 0.1–2.0	N/A	
Dosage of methadone (g/day)	39.41±20.45	N/A	

The duration of heroin usage means the period from the time of their initial heroin use to the time of their seeking medical attention.

^a^ Fisher's exact test.

*t*-test.^b^ Two sample

Neither the HDIs nor NCs had any history of neurological illness or head injury or had been diagnosed with schizophrenia or an affective disorder according to their past medical history. This study was approved by the Research Ethics Review Board of the Southern Medical University in Guangzhou of China. Informed written consent was obtained from each subject prior to the MRI scanning.

### Data acquisition

MRI data were obtained on a 1.5T Philips Achieva Nova Dual MR scanner in the Department of Medical Imaging, Guangdong No. 2 Provincial People's Hospital. The RS-fMRI data were obtained using a T2*-weighted gradient-echo echo-planar imaging (EPI) sequence with the following parameters, TR = 2000 ms, TE = 50 ms, flip angle = 90°, matrix = 64×64, FOV = 230×230 mm^2^, thickness/gap = 4.5/0 mm, 22 axial slices covering the whole brain, 240 volumes obtained in about 8 min. During the RS-fMRI scanning, all lights in the scanner room were switched off, and the subjects were instructed to close their eyes, to keep still, not to think systematically about anything, and not to fall asleep. In addition, we acquired 3D high resolution brain structural images using a T1-weighted 3D turbo-gradient-echo sequence (TR = 25 ms, TE = 4.1 ms, flip angle = 30°, matrix = 256×256, FOV = 230×230 mm^2^, thickness = 1.0 mm, and 160 sagittal slices).

### Data preprocessing

All the MRI data were processed using SPM8 (http://www.fil.ion.ucl.ac.uk/spm/) and DPARSF_V2.0 (http://www.restfmri.net/forum/index.php) [25]. For each subject, we first removed the first 10 volume images from the RS-fMRI data for scanner stabilization and for the subject's adaptation to the environment, leaving 230 volumes for further analysis. Then we performed slice timing to correct for the acquisition time delay between slices within the same TR, realignment to the first volume to correct the inter-TR head motions, spatial normalization to a standard MNI template and resampling to a voxel size of 3×3×3 mm^3^. No spatial smoothing was applied by following previous studies [26], [27], [28]. Finally, we performed band-pass filtering for each voxel in the frequency of 0.01–0.08 Hz to reduce low-frequency drift and high-frequency physiological noise. The RS-fMRI data for each subject were checked for head motion. No subject was excluded according to the criteria that the translation and rotation of head motion in any direction were not more than 1.5 mm or 1.5°.

### Network analysis

#### Network construction

In order to construct brain functional networks for each subject, we applied an automated anatomical labeling (AAL) atlas [29] to parcellate the brain into 90 regions of interest (ROIs) (45 in each hemisphere). The names of the ROIs and their corresponding abbreviations are listed in Table S2. The time series for each ROI was calculated by averaging the signals of all voxels within that region and by linearly regressing out the following nine nuisance covariates: three translation and three rotation head motion parameters and the white matter, cerebrospinal fluid (CSF), and global mean signals. For each subject, we obtained a 90×90 correlation matrix by calculating the Pearson's correlation coefficient in the residual time courses between all ROI-pairs. This matrix contained both negative and positive values, we used the absolute value of each element as the inter-regional functional connectivity by following previous studies [19], [26], [30], [31]. Finally, this correlation matrix was thresholded into a binarized matrix with a sparsity value (the ratio between total number of edges and the maximum possible number of edges in a network). By taking each ROI as a node and the functional connectivity as an edge, we obtained a 90×90 connectivity matrix for each subject and analyzed the topological organization of the whole-brain functional networks according to graph theory.

Clearly, the choice of a sparsity value has a major effect on the topological organization of networks [32], [33]. By setting a specific sparsity as the threshold, we were able to ensure that the brain functional networks corresponding to each subject contained the same number of edges. In order to balance the prominence of the small-world attribute with an appropriate level of sparseness in the networks for all subjects, we determined the range of sparsity according to the following criteria: 1) the averaged degree (total number of edges divided by *N*/2, with *N* = 90 here, denoting the number of nodes) over all nodes of each network was larger than log(*N*) [34], [35]; and 2) the small-worldness of the network for each subject was larger than 1.1 [33], [34]. Thus, we determined the range of sparsity (0.05≤s≤0.36) in which the network for each subject holds the small-worldness property. Using different threshold values over the range of 0.05≤s≤0.36 and intervals of 0.01, we set the connectivity matrix into a series of binaried connectivity matrices for each subject and calculated the topological properties. The subsequent network analysis was based on the series of binarized connectivity matrices for each subject.

#### Network parameters

We described the global topological properties of the brain functional networks by using the following seven global network parameters: the clustering coefficient (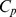), characteristic path length (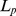), normalized clustering coefficient (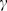), normalized characteristic path length (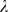), small-worldness (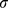), global efficiency (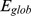), and local efficiency (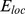). Their expression and detailed descriptions are listed in Table S3.

Two nodal centrality metrics, nodal degree (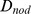) and nodal efficiency (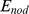), were used to describe the nodal properties of brain functional networks. Their expressions and descriptions are also presented in Table S3.

Instead of selecting a single sparsity threshold, we used the integrated network parameters over the range of sparsity to detect the between-group differences in the topological parameters of the brain functional networks. The integrated global parameters were given by [33]:
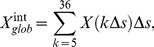
(1)where the sparsity interval 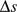 equals 0.01 and 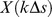 refers to any of the global parameters (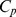, 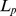, 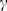, 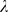, 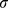, 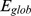, and 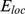) at a sparsity of 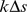. Similarly, the integrated nodal parameters can be calculated by [33]:
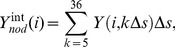
(2)where 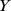 represents either nodal parameters (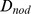, 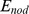) of node 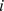 at a sparsity of *k*Δ*s*.

#### Hub identification

Hubs refer to highly connected nodes in the network [32]. Here, following the method used in previous studies [15], [36], we used nodal betweenness centrality (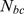) to determine the hub regions of the brain functional networks (for a detailed description, see Table S3. For each node, we first calculated its normalized nodal betweenness centrality as follows:
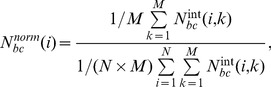
(3)where 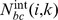 is the integrated nodal betweenness centrality of node 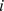 in the network of subject 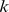, 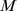 is the number of subjects in each group and 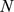 is the number of nodes (here 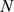 = 90). Nodes satisfying the criterion of 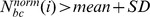 were considered to be the hubs of the brain functional networks [33]. Based on this criterion, we then identified the hubs of the brain functional networks separately for HDIs and NCs.

### Statistical analysis

#### Between-group differences

Two sample *t*-tests were performed to assess differences in age, duration of education, cigarette smoking, and head motions between the heroin addict group and the control group using SPSS (version 17.0). We used Fisher's exact test to estimate the difference in gender between the two groups (SPSS, version 17.0). Significant between-group differences were determined at *p*<0.05 (two-tailed).

A nonparametric permutation test [37] was performed to determine significant differences in each integrated network metric (five global parameters and two nodal centrality metrics) between the two groups. Briefly, for each network metric, we first calculated the between-group difference in the mean values. To obtain an empirical distribution of the difference, we then randomly reallocated all the values into two groups and recomputed the mean differences between the two randomized groups (10,000 permutations). The limits of the 95th percentile for each empirical distribution were used as the critical values for a two-tailed test of whether the observed group differences could occur by chance. To check the statistical power for the between-group comparisons in nodal metrics, we also estimated the effect sizes (Cohen *d*) according to Cohen's definition [38].

We used a network-based statistic (NBS) approach [39] to detect differences in the inter-nodal functional connections between the HDIs and NCs. In brief, a primary cluster-defining threshold was first used to identify suprathreshold connections for which the size (i.e., number of edges) of any connected components was then determined. A corrected *p*-value was calculated for each component using the null distribution of the maximally connected component size, which was derived empirically using a nonparametric permutation approach. The detailed descriptions are provided in Text S1.

#### Correlations between network parameters and duration of heroin addiction

We analyzed the correlation between each of the network parameters and the duration of heroin addiction in HDIs using a multiple linear regression. The significance levels were set at *p*<0.05 (two-tailed).

Although the two groups were statistically matched for age, the heroin group was an average of 5 years older than the control group. To control for any potential age-related effect, all of the above analyses were repeated after removing the confounding effect of age using a multiple linear regression.

## Results

### Demographic information

Statistical comparisons showed no significant differences in gender, age, duration of education, cigarette smoking, and head motions between the heroin group and the control group (Table 1).

### Global parameters

**Figure 1:**
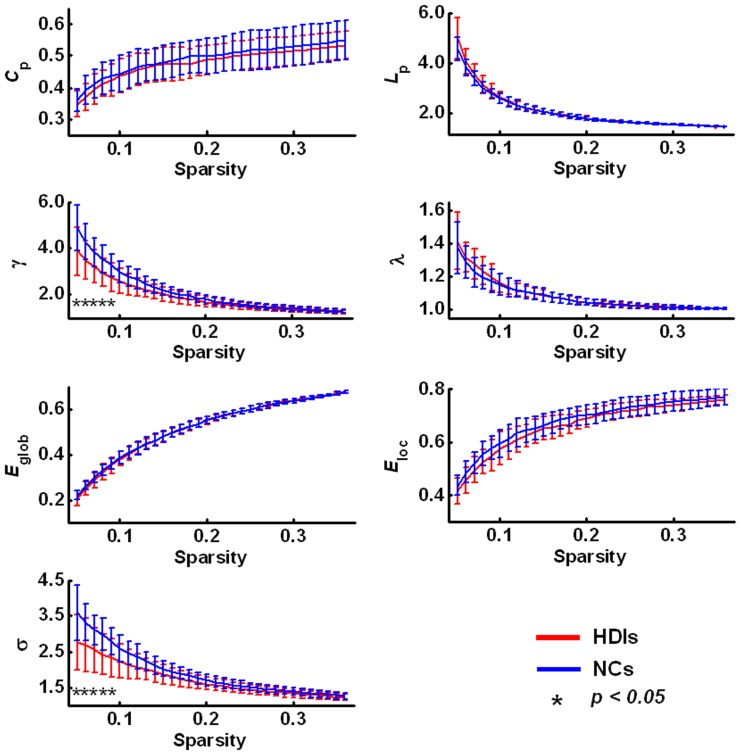
Global parameters of the brain functional networks for the heroin-dependent individuals (HDIs) and the normal controls (NCs) changing with the sparsity threshold. The error bar represents the standard deviation of a parameter at a given sparsity across all subjects. The symbol (*) means that significant between-group difference in the given parameter was detected (*p*<0.05). Except for the sparsity range of 0.05≤sparsity≤0.09, no statistically significant between-group differences were detected for other values of sparsity. 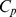, clustering coefficient; 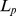, characteristic path length; 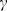, normalized clustering coefficient; 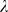, normalized shortest path length; 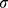, small-worldness; 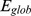, global efficiency; 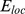, local efficiency.

Fig. 1 shows the plots of the global parameters (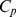, 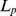, 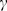, 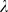, 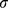, 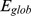, and 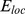) of the whole-brain functional networks changing with sparsity in both the HDIs and NCs. Fig. 1 also shows the comparisons for the values of 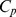, 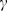, 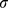, 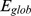, and 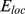 to be lower, but the values of 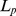 and 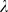 to be higher, in HDIs compared to NCs.

**Figure 2:**
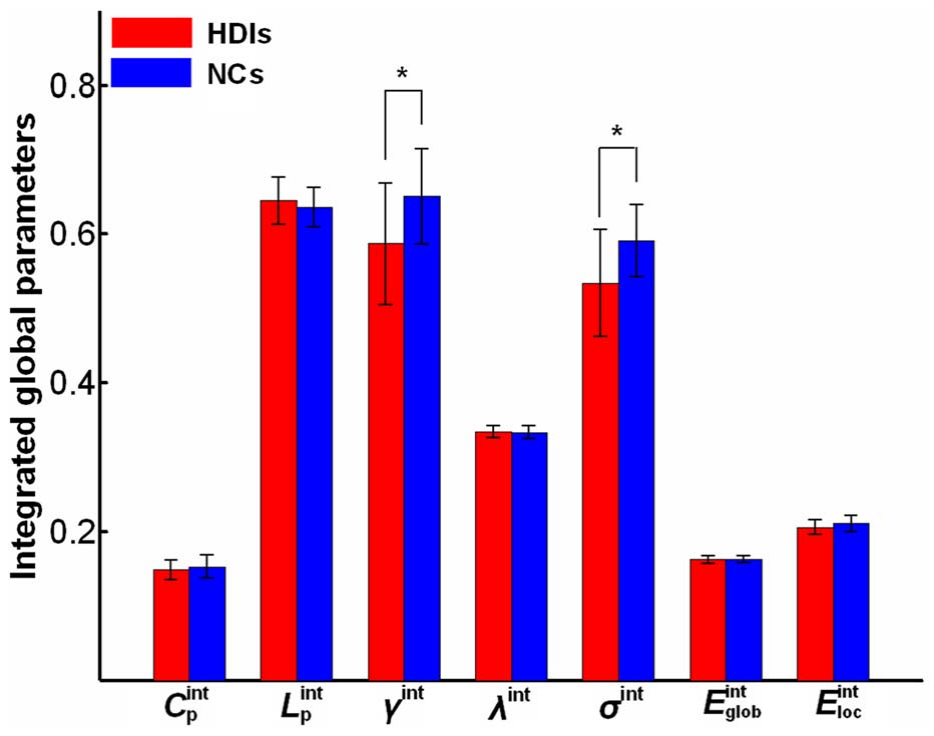
Bar plots of the differences in the integrated global topological parameters of brain functional networks between the heroin-dependent individuals (HDIs) and the normal controls (NCs). The symbol (*) indicates significant between-group differences in the integrated normalized clustering coefficient 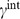 (*p* = 0.049) and integrated small-worldness 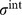 (*p* = 0.035). 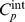, integrated clustering coefficient; 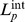, integrated characteristic path length; 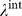, integrated normalized shortest path length; 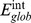, integrated global efficiency; 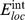, integrated local efficiency.

Fig. 2 shows statistical comparisons of the integrated global parameters between HDIs and NCs. The HDIs exhibited significantly lower values for integrated small-worldness 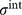 (*p* = 0.035) and the integrated normalized clustering coefficient 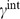 (*p* = 0.049) compared to the controls. However, we found no significant between-group difference in any of the integrated parameters 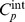, 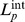, 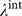, 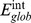, and 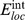.

### Nodal parameters

**Figure 3:**
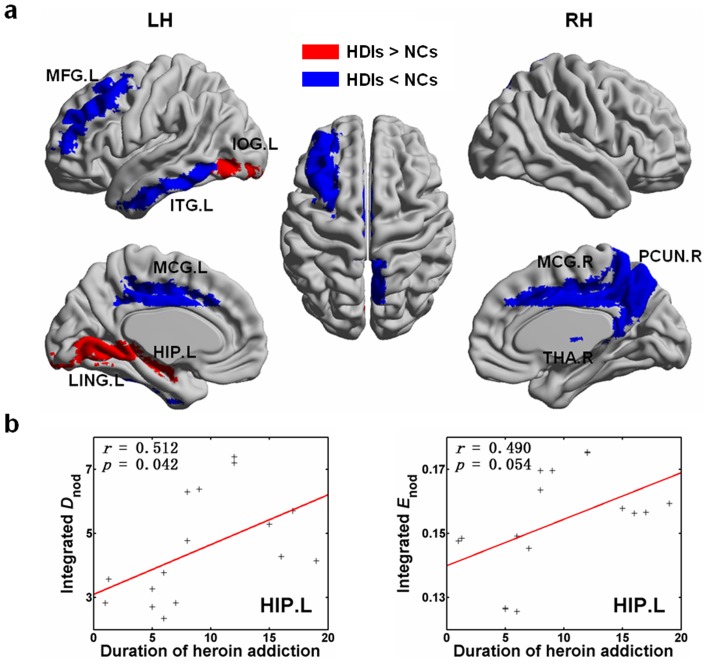
Brain regions exhibiting abnormal integrated nodal parameters of the brain functional networks and their relationship with the duration of heroin addiction in the heroin-dependent individuals (HDIs) compared to the normal controls (NCs). (a) Surface visualization of regions with abnormal nodal centralities using BrainNet Viewer (http://www.nitrc.org/projects/bnv/). Areas color-coded in red (blue) indicate the regions in which the values of nodal centralities corresponding to HDIs were higher (lower) than those of controls. See Table 2 for details. (b) Scatter plot of the integrated nodal parameters against the duration of heroin addiction. In the left hippocampus (HIP.L), we detected a significantly positive correlation between the integrated nodal degree and the duration of heroin addiction (*p* = 0.042) and a tendency toward a positive correlation between the integrated nodal efficiency and the duration of heroin addiction (*p* = 0.054) in HDIs. The abbreviations of regions are listed in Table S2.

Table 2 lists the brain regions that showed a significant difference in any of nodal centrality metrics (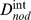 and 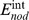) of the brain functional networks between HDIs and NCs (*p*<0.05, uncorrected). We found that in HDIs, the nodal centrality metrics were significantly decreased in six brain regions, the bilateral middle (dorsal) cingulate gyrus (MCG.L/R), left middle frontal gyrus (MFG.L), left inferior temporal gyrus (ITG.L), right precuneus (PCUN.R), and right thalamus (THA.R), most of which belong to the cognitive control network [40], [41], [42]; but significantly increased in three brain regions, the left hippocampus (HIP.L), left inferior occipital gyrus (IOG.L), and left lingual gyrus (LING.L). These regions were rendering plotted on a cortical surface map and are shown in Fig. 3a. The result of effect sizes presented in Table 2 indicated high statistical power of the between-group comparions in nodal parameters.

**Table 2:** Brain regions showing abnormal nodal centrality in the heroin-dependent individuals (HDIs) compared with the normal controls (NCs).

Regions	Classification	Mean (SD)				*p*-value (Cohen *d*)	
		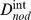		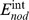		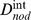	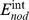
		HDIs	NCs	HDIs	NCs		
HDIs<NCs							
ITG.L	Association	3.50 (1.35)	5.23 (1.66)	0.15 (0.02)	0.16 (0.01)	0.0007 (1.2)	0.0005 (1.2)
MCG.L	Paralimbic	6.62 (1.99)	8.67 (1.99)	0.17 (0.02)	0.19 (0.01)	0.002 (1.0)	0.002 (1.0)
MCG.R	Paralimbic	6.63 (1.87)	8.08 (1.54)	0.18 (0.02)	0.19 (0.01)	0.004 (0.8)	0.006 (0.8)
THA.R	Subcortex	4.03 (2.34)	5.98 (2.18)	0.14 (0.03)	0.17 (0.02)	0.045 (0.9)	0.037 (0.9)
PCUN.R	Association	5.93 (1.93)	6.66 (1.99)	0.17 (0.02)	0.18 (0.02)	0.046 (0.4)	0.049 (0.4)
MFG.L	Association	5.43 (1.32)	6.58 (1.93)	0.17 (0.01)	0.18 (0.01)	—	0.048 (0.8)
HDIs>NCs							
LING.L	Association	8.11 (1.50)	6.19 (1.36)	0.18 (0.01)	0.17 (0.01)	0.001 (1.3)	0.005 (1.2)
IOG.L	Association	6.51 (1.66)	5.19 (1.68)	0.17 (0.01)	0.16 (0.01)	0.012 (0.8)	0.020 (0.7)
HIP.L	Subcortex	4.50 (1.61)	3.03 (1.59)	0.15 (0.02)	0.13 (0.03)	0.014 (0.9)	0.014 (0.9)

*p*<0.05 (uncorrected). The symbol ‘—’ indicates no significant between-group difference. 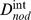 and 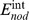 represent the integrated nodal degree and nodal efficiency, respectively. Cohen *d* indicates the value of effect size. The small, medium, and large levels of the effect size are 0.2, 0.5, and 0.8, respectively, according to Cohen's definition [38]. The threshold was

### Network hubs

The hub regions in the functional networks for HDIs and NCs are listed in Table S4. We found fourteen hubs in the brain functional networks of each subject group. Although the two groups had the identical number of hubs, the locations of the hubs were not completely the same. Eleven regions were shared hubs in the brain functional networks of both groups. We also found three hubs specific to HDIs, the left precuneus (PCUN.L), left postcentral gyrus (PoCG.L), and right middle frontal gyrus (MFG.R), and three hubs specific to the controls, the left middle frontal gyrus (MFG.L), right precuneus (PCUN.R), and temporal pole (TPOsup.R). We noticed that most of the shared hub regions (nine hubs) were located in the association cortices, suggesting that they had important functional roles in information transfer [14].

### Functional connectivity

**Figure 4:**
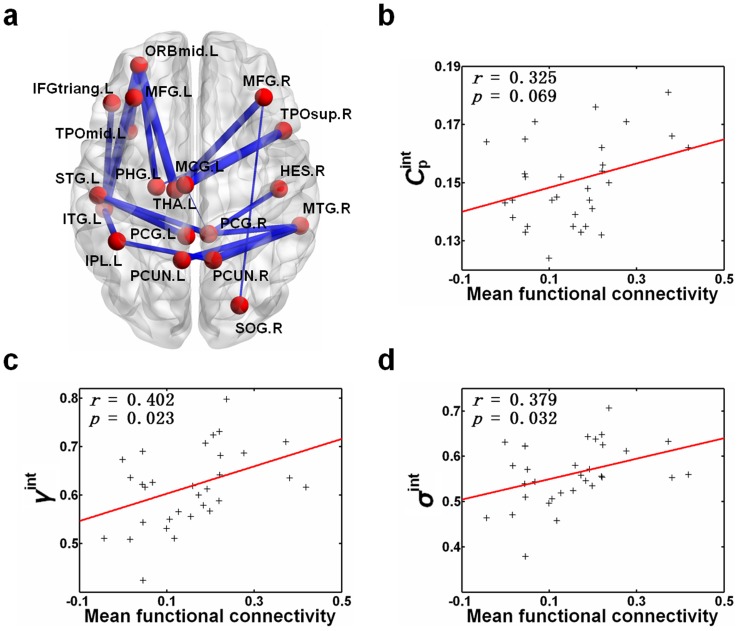
The connected subnetwork showing decreased functional connections in the heroin-dependent individuals (HDIs) compared to the normal controls (NCs). (a) Visualization of decreased functional connections related to heroin addiction using BrainNet Viewer (http://www.nitrc.org/projects/bnv/). The width of the line indicates the *t*-value of the connection comparisons between the two groups. A single, connected subnetwork containing 19 nodes and 19 connections was determined using the network-based statistic (NBS) method. (b) Scatter plot of the integrated clustering coefficient, 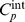, against the mean functional connectivity of this connected subnetwork averaged over all subjects. (c) Same as (b) but for the integrated normalized clustering coefficient, 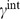. (d) Same as (b) but for integrated small-worldness, 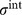. The abbreviations of the regions are listed in Table S2.

We utilized the NBS method to identify a single connected subnetwork with 19 regions and 19 connections, which was significantly altered in the HDIs compared to NCs (*p*<0.001, corrected) (Fig. 4, Table S5). We noticed that the connections in this single connected subnetwork are primarily long-distance connections linking different brain lobes. Within this subnetwork, all connections exhibited statistically significantly decreased values in HDIs (Table S5). We found that the mean connectivity value of this subnetwork correlated positively with three integrated global parameters, 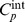 (*r* = 0.325, *p* = 0.069, marginally significant), 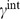 (*r* = 0.402, *p* = 0.023), and 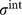 (*r* = 0.379, *p* = 0.032) (Fig. 4).

### Correlations between network parameters and duration of heroin addiction

No significant correlations (*p*>0.05) were found between the integrated global parameters and the duration of heroin addiction as well as between the connections shown in Fig. 4a and the duration of heroin addiction. For the brain regions listed in Table 2, we found that the integrated degree (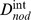) of the HIP.L showed a significantly positive correlation (*p* = 0.042), while the integrated nodal efficiency (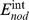) showed a marginally significantly positive correlation (*p* = 0.054) with the duration of heroin addiction (Fig. 3b).

## Discussion

In this study, using graph theory analysis, we constructed the functional networks, analyzed the network topological parameters, and compared the differences in these parameters of the brain functional networks between HDIs and NCs. The main findings are as follows: (1) at the global level, the heroin group showed significant decreases in the normalized clustering coefficient and in small-worldness; (2) at the nodal level, we detected significantly decreased nodal centralities primarily in regions of the cognitive control network but significant increases primarily in the HIP.L in HDIs; (3) at the connectivity level, we found a single connected subnetwork which showed significantly decreased connections in the heroin group. These findings may contribute to understanding the disrupted topological organization of whole-brain functional networks in HDIs.

### Global parameters

The human brain is widely believed to be a complex system that requires a suitable balance between local specialization and global integration of the brain's functional activities [43]. Functional segregation and integration are two fundamental organizing principles for the human brain, a concept which is supported by the model of a small-world network characterized by a high local clustering coefficient and the shortest path length [32]. Small-world properties enable a network to maintain highly effective, specialized modular information processing as well as rapid global information transfer [44]. As has been found in previous studies of human brain functional networks [16], [17], [18], [20], [33], in this study, the whole-brain functional networks of both HDIs and NCs conserved small-worldness.

In this study, we found alterations in the global parameters of the brain functional networks of HDIs compared to NCs. Statistical analysis revealed a decreased normalized clustering coefficient in the HDIs. The normalized clustering coefficient is one of the indices that can characterize how brain networks shift to either a regular or a random network [45]. The decreased clustering coefficient in HDIs indicated that their brain functional networks may shift toward random organization. Previous studies [9], [46], [47] have suggested heroin users showed poor performance in decision making tasks compared to healthy participants. Shift toward random organization in functional network may be related with randomized decision making in HDIs. In addition, we also detected decreased small-worldness in HDIs, suggesting the topological organization in the whole-brain functional networks of HDIs was less optimal than that of the controls. Those decreases in the global network parameters in HDIs may have resulted from the decreased functional connections in a subnetwork (Fig. 4, Table S5) [19]. These connections enable spatially remote brain regions to communicate with each other and strengthen the functional integration of the brain [48]. Finding that these decreases may indicate that the brain functional integration in HDIs is disrupted.

### Nodal parameters

Besides of the decreased global parameters, we also found decreased nodal centralities (integrated nodal degree and nodal efficiency) in several regions in HDIs, including the MCG.L/R, MFG.L, and PCUN.R. These regions are thought to be involved in the cognitive control network [40], [41], [42]. Previous studies have suggested drug addiction individuals exhibited deficits in neural systems associated with cognitive control [12], [49], [50], [51]. In a task-fMRI study, Kaufman et al. [49] found the dorsal cingulate cortex was less responsive during successful No-Go inhibitions in cocaine users, suggesting the drug-related dysfunction of cognitive control. Using resting-state fMRI, Ma et al. [12] found that heroin users showed reduced functional connectivity within the circuit of cognitive control, indicating the weakened strength of control in the addictive state. Recently, Liu et al. [50] studied heroin users using diffusion tensor imaging and reported that heroin users showed reduced white matter integrity in the frontal and cingulate cortex, which suggested diminished cognitive control upon craving and motivation in heroin users. Thus, our findings of decreased nodal centralities in the cognitive control regions in HDIs provided further evidence that the function of cognitive control is weakened in drug addiction [52].

Interestingly, we found the MFG and PCUN were hub regions for HDIs and NCs, but located in contralateral hemisphere, i.e., MFG.R and PCUN.L were hubs of HDIs, while MFG.L and PCUN.R were hubs of NCs. This finding may reflect the existing compensatory mechanism or neuroadaptation in the addiction brain [53], [54], [55]. For example, Jager et al. [54] found that the adolescent cannabis users showed excessive activity in the prefrontal regions during a novel task, suggesting functional compensation. Also Kanayama et al. [55] reported that the cannabis users might call upon additional brain regions not typically used for spatial working memory (such as regions in the basal ganglia) to compensate for the deficits in spatial working memory. In the present study, the brain functional networks of HDIs may need to enhance the function of MFG.R and PCUN.L to compensate for impaired function of MFG.L and PCUN.R due to their decreased nodal centralities in HDIs.

We also found that the HDIs showed increased nodal centralities in the HIP.L compared to NCs, a finding which was consistent with several previous studies [11], [52], [56]. Using graph theory analysis, Liu et al. [11] suggested that the hippocampus had a higher nodal degree in the brain of chronic heroin users. Ma et al. [56] found that heroin users showed increased functional connectivity in the hippocampus compared to controls. Baler and Volkow [52] demonstrated that the memory/learning circuit related to drug addiction is primarily located in the amygdala and hippocampus. In fact, the hippocampus is the main brain region involved in memory and learning [57], and is thought to strengthen the learning of drug-related cues which leads to drug-seeking behaviors [58]. Therefore, an increase in nodal centrality in the HIP.L may excite the expectation of the drug in HDIs.

Moreover, the integrated nodal degree and the nodal efficiency were positively correlated with the duration of heroin addiction, but only in the HIP.L. This indicated that longer the heroin use, the higher the nodal centrality of the HIP.L. Thus, the disrupted topology properties in the hippocampus may indicate that the pattern of relapse to drug-seeking behaviors that is commonly seen in HDIs is driven by abnormal memory processing.

Notably, the disrupted nodal topology can also be interpreted from the perspective of physiological aspect. Previous studies [59], [60], [61], [62], [63] have suggested that the effects of opioid drugs on the brain might depend on the opioid receptor density. The frontal cortex and cingulate cortex (anterior and middle) have the high opiate receptor-binding potentials [64] and have been reported to be commonly affected by different opoioid drugs, such as cocaine [59], nicotine [61], morphine [62], [65] and remifentanil [63], [66]. Thus, the current findings of decreased nodal centralities in the left middle frontal cortex and bilateral middle cingulate cortex in HDIs may reflect an outcome of disrupted opioidergic modulation. Actually, we cannot attribute all altered nodal centralities to opioid receptor. In current study, we also found heroin affected nodal centrality in the left hippocampus which has not been reported to include high opioid receptor. This is in line with several task and resting-state pharmacological fMRI studies [62], [65], which reported that morphine affected functional topography of hippocampus in healthy volunteers. We noticed that no change of nodal centrality has been detected in this study in at least several areas, the insula, thalamus, amygdala, and putamen, though these regions are more susceptible to the high opioid receptor [64]. These suggest that the findings of abnormal nodal centralities in HDIs partly reflect the opioid receptor distribution.

### Limitations

Several limitations need to be addressed. First, due to the cross-sectional nature of this study, we can only infer that the network properties of the brain functional networks of heroin addicts are disrupted. We are not able to determine the precise relationship between heroin abuse and abnormalities of the network parameters. Second, the nodal centrality results could not survive when we adopted multiple comparisons (FDR and FEW corrections), meaning this should be considered as an exploratory analysis. To increase the statistical power, the findings need replication with a larger sample of subjects or a limited number of selected ROIs. Third, the HDIs received methadone treatment at the time of the fMRI study which might affect the brain spontaneous activity [67], [68] and the topological properties of functional network in the present study. Therefore, in the future, we should design a more rigorous experiment to exclude the effect of methadone. Fourth, we could not completely eliminate the effects of physiologic noise due to the low sampling rate (TR = 2 s), which can cause respiratory and cardiac fluctuations to impact the fMRI time series, even though a 0.01–0.08 Hz band-pass filter was used to reduce this effect. Finally, we only estimated the relations between the network properties and the duration of heroin addiction. Whether brain functional network properties are related with other clinical variables including decision-making behavior, impulsivity and consequences on daily life should be explored in the future.

In summary, we investigated the whole-brain functional network in HDIs using resting-state fMRI and a graph theory method. We found that compared to the normal controls, the whole-brain functional networks in HDIs may shift toward random organization, as indicated by a lower normalized clustering coefficient and lessened small-worldness. We also found that the nodal properties were disrupted, especially in regions of cognitive control network in HDIs. Our study indicated disruptions in the whole-brain functional networks of HDIs, findings which may be helpful for better understanding the mechanisms underlying heroin addiction.

## Supporting Information

Text S1 **Network-Based-Statistic Analysis (NBS).**
(DOC)

Table S1 **The detailed clinical description for each heroin-dependent individual (HDIs).**
(DOC)

Table S2 **The names and the corresponding abbreviations of the regions of interest (ROIs).**
(DOC)

Table S3 **The mathematical definitions and descriptions of global metrics and nodal metrics.**
(DOC)

Table S4 **Hub regions of the brain functional networks detected in the heroin-dependent individuals (HDIs) and normal controls (NCs).**
(DOC)

Table S5 **Decreased functional connections in the heroin-dependent individuals (HDIs) as compared to the normal controls (NCs).**
(DOC)

## References

[1] Volkow ND, Li TK (2004) Drug addiction: the neurobiology of behaviour gone awry. Nat Rev Neurosci 5: 963–970. 15550951 10.1038/nrn1539

[2] Tang YL, Zhao D, Zhao C, Cubells JF (2006) Opiate addiction in China: current situation and treatments. Addiction 101: 657–665. 16669899 10.1111/j.1360-0443.2006.01367.x

[3] Dackis CA, Gold MS (1985) New concepts in cocaine addiction: the dopamine depletion hypothesis. Neurosci Biobehav Rev 9: 469–477. 2999657 10.1016/0149-7634(85)90022-3

[4] Goldstein RZ, Volkow ND (2002) Drug addiction and its underlying neurobiological basis: neuroimaging evidence for the involvement of the frontal cortex. Am J Psychiatry 159: 1642–1652. 12359667 10.1176/appi.ajp.159.10.1642 PMC1201373

[5] Lee TM, Zhou WH, Luo XJ, Yuen KS, Ruan XZ, et al (2005) Neural activity associated with cognitive regulation in heroin users: a fMRI study. Neurosci Lett 382: 211–216. 15925092 10.1016/j.neulet.2005.03.053

[6] Anand A, Li Y, Wang Y, Lowe MJ, Dzemidzic M (2009) Resting state corticolimbic connectivity abnormalities in unmedicated bipolar disorder and unipolar depression. Psychiatry Res 171: 189–198. 19230623 10.1016/j.pscychresns.2008.03.012 PMC3001251

[7] Greicius M (2008) Resting-state functional connectivity in neuropsychiatric disorders. Curr Opin Neurol 21: 424–430. 18607202 10.1097/WCO.0b013e328306f2c5

[8] Lynall ME, Bassett DS, Kerwin R, McKenna PJ, Kitzbichler M, et al (2010) Functional connectivity and brain networks in schizophrenia. J Neurosci 30: 9477–9487. 20631176 10.1523/JNEUROSCI.0333-10.2010 PMC2914251

[9] Qiu YW, Han LJ, Lv XF, Jiang GH, Tian JZ, et al (2011) Regional homogeneity changes in heroin-dependent individuals: resting-state functional MR imaging study. Radiology 261: 551–559. 21875854 10.1148/radiol.11102466

[10] Jiang GH, Qiu YW, Zhang XL, Han LJ, Lv XF, et al (2011) Amplitude low-frequency oscillation abnormalities in the heroin users: a resting state fMRI study. NeuroImage 57: 149–154. 21515385 10.1016/j.neuroimage.2011.04.004

[11] Liu J, Liang J, Qin W, Tian J, Yuan K, et al (2009) Dysfunctional connectivity patterns in chronic heroin users: an fMRI study. Neurosci Lett 460: 72–77. 19450664 10.1016/j.neulet.2009.05.038

[12] Ma N, Liu Y, Li N, Wang CX, Zhang H, et al (2010) Addiction related alteration in resting-state brain connectivity. NeuroImage 49: 738–744. 19703568 10.1016/j.neuroimage.2009.08.037 PMC2764798

[13] Zhang Y, Tian J, Yuan K, Liu P, Zhuo L, et al (2011) Distinct resting-state brain activities in heroin-dependent individuals. Brain Res 1402: 46–53. 21669407 10.1016/j.brainres.2011.05.054

[14] He Y, Chen ZJ, Evans AC (2007) Small-world anatomical networks in the human brain revealed by cortical thickness from MRI. Cereb Cortex 17: 2407–2419. 17204824 10.1093/cercor/bhl149

[15] Yan C, Gong G, Wang J, Wang D, Liu D, et al (2011) Sex- and brain size-related small-world structural cortical networks in young adults: a DTI tractography study. Cereb Cortex 21: 449–458. 20562318 10.1093/cercor/bhq111

[16] Supekar K, Menon V, Rubin D, Musen M, Greicius MD (2008) Network analysis of intrinsic functional brain connectivity in Alzheimer's disease. PLoS Comput Biol 4: e1000100. 18584043 10.1371/journal.pcbi.1000100 PMC2435273

[17] Zhang Z, Liao W, Chen H, Mantini D, Ding JR, et al (2011) Altered functional-structural coupling of large-scale brain networks in idiopathic generalized epilepsy. Brain 134: 2912–2928. 21975588 10.1093/brain/awr223

[18] Liao W, Zhang Z, Pan Z, Mantini D, Ding J, et al (2010) Altered functional connectivity and small-world in mesial temporal lobe epilepsy. PLoS ONE 5: e8525. 20072616 10.1371/journal.pone.0008525 PMC2799523

[19] Zhang J, Wang J, Wu Q, Kuang W, Huang X, et al (2011) Disrupted brain connectivity networks in drug-naive, first-episode major depressive disorder. Biol Psychiatry 70: 334–342. 21791259 10.1016/j.biopsych.2011.05.018

[20] Liu Y, Liang M, Zhou Y, He Y, Hao Y, et al (2008) Disrupted small-world networks in schizophrenia. Brain 131: 945–961. 18299296 10.1093/brain/awn018

[21] Rubinov M, Knock SA, Stam CJ, Micheloyannis S, Harris AW, et al (2009) Small-world properties of nonlinear brain activity in schizophrenia. Hum Brain Mapp 30: 403–416. 18072237 10.1002/hbm.20517 PMC6871165

[22] Yuan K, Qin W, Liu J, Guo Q, Dong M, et al (2010) Altered small-world brain functional networks and duration of heroin use in male abstinent heroin-dependent individuals. Neurosci Lett 477: 37–42. 20417253 10.1016/j.neulet.2010.04.032

[23] Ersche KD, Fletcher PC, Roiser JP, Fryer TD, London M, et al (2006) Differences in orbitofrontal activation during decision-making between methadone-maintained opiate users, heroin users and healthy volunteers. Psychopharmacology (Berl) 188: 364–373. 16953385 10.1007/s00213-006-0515-z PMC1903380

[24] Yuan K, Qin W, Dong M, Liu J, Liu P, et al (2010) Combining spatial and temporal information to explore resting-state networks changes in abstinent heroin-dependent individuals. Neuroscience Letters 475: 20–24. 20302912 10.1016/j.neulet.2010.03.033

[25] Yan C-G, Zang Y-F (2010) DPARSF: A MATLAB Toolbox for “Pipeline” Data Analysis of Resting-State fMRI. Front Syst Neurosci 4: 13. 20577591 10.3389/fnsys.2010.00013 PMC2889691

[26] Braun U, Plichta MM, Esslinger C, Sauer C, Haddad L, et al (2012) Test-retest reliability of resting-state connectivity network characteristics using fMRI and graph theoretical measures. NeuroImage 59: 1404–1412. 21888983 10.1016/j.neuroimage.2011.08.044

[27] Wang J, Wang L, Zang Y, Yang H, Tang H, et al (2009) Parcellation-dependent small-world brain functional networks: A resting-state fMRI study. Human Brain Mapping 30: 1511–1523. 18649353 10.1002/hbm.20623 PMC6870680

[28] Achard S, Bullmore E (2007) Efficiency and cost of economical brain functional networks. PLoS Comput Biol 3: e17. 17274684 10.1371/journal.pcbi.0030017 PMC1794324

[29] Tzourio-Mazoyer N, Landeau B, Papathanassiou D, Crivello F, Etard O, et al (2002) Automated anatomical labeling of activations in SPM using a macroscopic anatomical parcellation of the MNI MRI single-subject brain. NeuroImage 15: 273–289. 11771995 10.1006/nimg.2001.0978

[30] Wang JH, Zuo XN, Gohel S, Milham MP, Biswal BB, et al (2011) Graph theoretical analysis of functional brain networks: test-retest evaluation on short- and long-term resting-state functional MRI data. PLoS ONE 6: e21976. 21818285 10.1371/journal.pone.0021976 PMC3139595

[31] Bassett DS, Bullmore E, Verchinski BA, Mattay VS, Weinberger DR, et al (2008) Hierarchical organization of human cortical networks in health and schizophrenia. J Neurosci 28: 9239–9248. 18784304 10.1523/JNEUROSCI.1929-08.2008 PMC2878961

[32] Rubinov M, Sporns O (2010) Complex network measures of brain connectivity: uses and interpretations. NeuroImage 52: 1059–1069. 19819337 10.1016/j.neuroimage.2009.10.003

[33] Tian L, Wang J, Yan C, He Y (2011) Hemisphere- and gender-related differences in small-world brain networks: a resting-state functional MRI study. NeuroImage 54: 191–202. 20688177 10.1016/j.neuroimage.2010.07.066

[34] Watts DJ, Strogatz SH (1998) Collective dynamics of ‘small-world’ networks. Nature 393: 440–442. 9623998 10.1038/30918

[35] Fornito A, Zalesky A, Bullmore ET (2010) Network scaling effects in graph analytic studies of human resting-state FMRI data. Front Syst Neurosci 4: 22. 20592949 10.3389/fnsys.2010.00022 PMC2893703

[36] He Y, Dagher A, Chen Z, Charil A, Zijdenbos A, et al (2009) Impaired small-world efficiency in structural cortical networks in multiple sclerosis associated with white matter lesion load. Brain 132: 3366–3379. 19439423 10.1093/brain/awp089 PMC2792366

[37] Bullmore ET, Suckling J, Overmeyer S, Rabe-Hesketh S, Taylor E, et al (1999) Global, voxel, and cluster tests, by theory and permutation, for a difference between two groups of structural MR images of the brain. IEEE Trans Med Imaging 18: 32–42. 10193695 10.1109/42.750253

[38] Cohen J (1992) A power primer. Psychol Bull 112: 155–159. 19565683 10.1037//0033-2909.112.1.155

[39] Zalesky A, Fornito A, Bullmore ET (2010) Network-based statistic: identifying differences in brain networks. NeuroImage 53: 1197–1207. 20600983 10.1016/j.neuroimage.2010.06.041

[40] Spreng RN, Schacter DL (2012) Default network modulation and large-scale network interactivity in healthy young and old adults. Cereb Cortex 22: 2610–2621. 22128194 10.1093/cercor/bhr339 PMC3464415

[41] Ridderinkhof KR, Ullsperger M, Crone EA, Nieuwenhuis S (2004) The role of the medial frontal cortex in cognitive control. Science 306: 443–447. 15486290 10.1126/science.1100301

[42] Vincent JL, Kahn I, Snyder AZ, Raichle ME, Buckner RL (2008) Evidence for a frontoparietal control system revealed by intrinsic functional connectivity. J Neurophysiol 100: 3328–3342. 18799601 10.1152/jn.90355.2008 PMC2604839

[43] Tononi G, Edelman GM, Sporns O (1998) Complexity and coherency: integrating information in the brain. Trends Cogn Sci 2: 474–484. 21227298 10.1016/s1364-6613(98)01259-5

[44] Kaiser M, Hilgetag CC (2006) Nonoptimal component placement, but short processing paths, due to long-distance projections in neural systems. PLoS Comput Biol 2: e95. 16848638 10.1371/journal.pcbi.0020095 PMC1513269

[45] He Y, Evans A (2010) Graph theoretical modeling of brain connectivity. Curr Opin Neurol 23: 341–350. 20581686 10.1097/WCO.0b013e32833aa567

[46] Li X, Zhang F, Zhou Y, Zhang M, Wang X, et al (2013) Decision-making deficits are still present in heroin abusers after short- to long-term abstinence. Drug Alcohol Depend 130: 61–67. 23131777 10.1016/j.drugalcdep.2012.10.012

[47] Vassileva J, Petkova P, Georgiev S, Martin EM, Tersiyski R, et al (2007) Impaired decision-making in psychopathic heroin addicts. Drug Alcohol Depend 86: 287–289. 16930861 10.1016/j.drugalcdep.2006.06.015

[48] Dosenbach NU, Nardos B, Cohen AL, Fair DA, Power JD, et al (2010) Prediction of individual brain maturity using fMRI. Science 329: 1358–1361. 20829489 10.1126/science.1194144 PMC3135376

[49] Kaufman JN, Ross TJ, Stein EA, Garavan H (2003) Cingulate hypoactivity in cocaine users during a GO-NOGO task as revealed by event-related functional magnetic resonance imaging. J Neurosci 23: 7839–7843. 12944513 10.1523/JNEUROSCI.23-21-07839.2003 PMC6740597

[50] Liu H, Li L, Hao Y, Cao D, Xu L, et al (2008) Disrupted white matter integrity in heroin dependence: a controlled study utilizing diffusion tensor imaging. Am J Drug Alcohol Abuse 34: 562–575. 18720268 10.1080/00952990802295238

[51] Sutherland MT, McHugh MJ, Pariyadath V, Stein EA (2012) Resting state functional connectivity in addiction: Lessons learned and a road ahead. NeuroImage 62: 2281–2295. 22326834 10.1016/j.neuroimage.2012.01.117 PMC3401637

[52] Baler RD, Volkow ND (2006) Drug addiction: the neurobiology of disrupted self-control. Trends in Molecular Medicine 12: 559–566. 17070107 10.1016/j.molmed.2006.10.005

[53] Lopez-Larson MP, Bogorodzki P, Rogowska J, McGlade E, King JB, et al (2011) Altered prefrontal and insular cortical thickness in adolescent marijuana users. Behav Brain Res 220: 164–172. 21310189 10.1016/j.bbr.2011.02.001 PMC3073407

[54] Jager G, Block RI, Luijten M, Ramsey NF (2010) Cannabis use and memory brain function in adolescent boys: a cross-sectional multicenter functional magnetic resonance imaging study. J Am Acad Child Adolesc Psychiatry 49: 561–572, 572 e561–563. 20494266 10.1016/j.jaac.2010.02.001 PMC2918244

[55] Kanayama G, Rogowska J, Pope HG, Gruber SA, Yurgelun-Todd DA (2004) Spatial working memory in heavy cannabis users: a functional magnetic resonance imaging study. Psychopharmacology (Berl) 176: 239–247. 15205869 10.1007/s00213-004-1885-8

[56] Ma N, Liu Y, Fu XM, Li N, Wang CX, et al (2011) Abnormal brain default-mode network functional connectivity in drug addicts. PLoS ONE 6: e16560. 21298074 10.1371/journal.pone.0016560 PMC3027699

[57] Riedel G, Micheau J (2001) Function of the hippocampus in memory formation: desperately seeking resolution. Prog Neuropsychopharmacol Biol Psychiatry 25: 835–853. 11383980 10.1016/s0278-5846(01)00153-1

[58] Robbins T, Ersche K, Everitt B (2008) Drug addiction and the memory systems of the brain. Ann N Y Acad Sci 1141: 1–21. 18991949 10.1196/annals.1441.020

[59] Gorelick DA, Kim YK, Bencherif B, Boyd SJ, Nelson R, et al (2005) Imaging brain mu-opioid receptors in abstinent cocaine users: time course and relation to cocaine craving. Biol Psychiatry 57: 1573–1582. 15953495 10.1016/j.biopsych.2005.02.026

[60] Henriksen G, Willoch F (2008) Imaging of opioid receptors in the central nervous system. Brain 131: 1171–1196. 18048446 10.1093/brain/awm255 PMC2367693

[61] Scott DJ, Domino EF, Heitzeg MM, Koeppe RA, Ni L, et al (2007) Smoking modulation of mu-opioid and dopamine D2 receptor-mediated neurotransmission in humans. Neuropsychopharmacology 32: 450–457. 17091130 10.1038/sj.npp.1301238

[62] Becerra L, Harter K, Gonzalez RG, Borsook D (2006) Functional magnetic resonance imaging measures of the effects of morphine on central nervous system circuitry in opioid-naive healthy volunteers. Anesth Analg 103: 208–216. 16790655 10.1213/01.ane.0000221457.71536.e0

[63] Pattinson KT, Governo RJ, MacIntosh BJ, Russell EC, Corfield DR, et al (2009) Opioids depress cortical centers responsible for the volitional control of respiration. J Neurosci 29: 8177–8186. 19553457 10.1523/JNEUROSCI.1375-09.2009 PMC6666048

[64] Baumgartner U, Buchholz HG, Bellosevich A, Magerl W, Siessmeier T, et al (2006) High opiate receptor binding potential in the human lateral pain system. NeuroImage 30: 692–699. 16337817 10.1016/j.neuroimage.2005.10.033

[65] Khalili-Mahani N, Zoethout RM, Beckmann CF, Baerends E, de Kam ML, et al (2012) Effects of morphine and alcohol on functional brain connectivity during “resting state”: a placebo-controlled crossover study in healthy young men. Hum Brain Mapp 33: 1003–1018. 21391283 10.1002/hbm.21265 PMC6870105

[66] MacIntosh BJ, Pattinson KT, Gallichan D, Ahmad I, Miller KL, et al (2008) Measuring the effects of remifentanil on cerebral blood flow and arterial arrival time using 3D GRASE MRI with pulsed arterial spin labelling. J Cereb Blood Flow Metab 28: 1514–1522. 18506198 10.1038/jcbfm.2008.46

[67] Wang Y, Li W, Li Q, Yang W, Zhu J, et al (2011) White matter impairment in heroin addicts undergoing methadone maintenance treatment and prolonged abstinence: a preliminary DTI study. Neurosci Lett 494: 49–53. 21362458 10.1016/j.neulet.2011.02.053

[68] Prosser J, London ED, Galynker II (2009) Sustained attention in patients receiving and abstinent following methadone maintenance treatment for opiate dependence: performance and neuroimaging results. Drug Alcohol Depend 104: 228–240. 19608356 10.1016/j.drugalcdep.2009.04.022

