# Supplementary material for: Disrupted Topological Organization in Whole-Brain Functional Networks of Heroin-Dependent Individuals: A Resting-State fMRI Study
Source: PLoS One. 2013 Dec 17;8(12):e82715. doi: 10.1371/journal.pone.0082715 (PMC3866189; doi:10.1371/journal.pone.0082715)
Supplement: Text S1 — Network-Based-Statistic Analysis (NBS). (DOC) [file pone.0082715.s001.doc]

**Network-Based-Statistic Analysis (NBS)**

To localize specific pairs of brain regions in which functional connectivity was altered in heroin-dependent individuals (HDIs), we applied the network-based statistic (NBS) approach . First, we detected significant nonzero connections (*p* < 0.05, uncorrected) in Fisher’s transformed correlation matrices for each group by performing multiple one-sample *t*-tests. Next, we combined the nonzero connections within either the HDIs or the normal controls (NCs) into a connection mask. At last, we conducted the NBS approach within the mask. After applied a primary threshold (*t* = 1.697, *p* = 0.05) to a *t*-statistic (two-sample *t*-test) for each link, we defined a set of suprathreshold links for which any connected components and their size (number of links) could be determined.

To estimate the significance for each component, we derived the null distribution of the connected component size empirically using a nonparametric permutation approach (1,000 times). For each permutation, all subjects were randomly reallocated to two groups and the *t*-statistic was computed independently for each link. We selected the same threshold (*t* = 1.697) to generate suprathreshold links for which the maximal connected component size was recorded. For a connected component of size *M* found in the right grouping of NCs and HDIs, we determined the corrected *p*-value by calculating the proportion of the 1,000 permutations for which the maximal connected component was larger than *M*. In the calculations, the effect of age was removed by a regression analysis before the statistical analysis of the functional connections. For a detailed description of the NBS approach, please see the study by Zalesky, Fornito et al. .

**References:**

1. Zalesky A, Fornito A, Bullmore E (2010) Network-based statistic: Identifying differences in brain networks. Neuroimage 53: 1197-1207.
