# Supplementary material for: Disrupted Topological Organization in Whole-Brain Functional Networks of Heroin-Dependent Individuals: A Resting-State fMRI Study
Source: PLoS One. 2013 Dec 17;8(12):e82715. doi: 10.1371/journal.pone.0082715 (PMC3866189; doi:10.1371/journal.pone.0082715)
Supplement: Table S1 — The detailed clinical description for each heroin-dependent individual (HDIs). (DOC) [file pone.0082715.s002.doc]

**Table S1.** The detailed clinical description for each heroin-dependent individual (HDIs).

| Subject | Duration of heroin addiction (years) | Heroin dosage (g/day) | Dosage of methadone (g/day) | Nicotine (No. cigarettes  /day) |
| --- | --- | --- | --- | --- |
| 1 | 12 | 0.8 | 30 | 20 |
| 2 | 12 | 1.0 | 60 | 10 |
| 3 | 5 | 0.5 | 20 | 10 |
| 4 | 8 | 0.5 | 0 | 20 |
| 5 | 15 | 0.3 | 40 | 20 |
| 6 | 19 | 0.5 | 60 | 10 |
| 7 | 1.3 | 2.0 | 50 | 10 |
| 8 | 6 | 2.0 | 40 | 0 |
| 9 | 7 | 0.5 | 40 | 20 |
| 10 | 6 | 0.3 | 40 | 20 |
| 11 | 6 | 1.0 | 60 | 0 |
| 12 | 8 | 0.3 | 0 | 40 |
| 13 | 5 | 1.0 | 60 | 40 |
| 14 | 17 | 1.0 | 60 | 20 |
| 15 | 9 | 0.1 | 20 | 20 |
| 16 | 1 | 1.0 | 30 | 40 |
| 17 | 16 | 1.0 | 60 | 20 |

All HDIs have the withdrawal symptoms such as anxiety, angry, sleeplessness, and constipation.
