# Supplementary material for: Disrupted Topological Organization in Whole-Brain Functional Networks of Heroin-Dependent Individuals: A Resting-State fMRI Study
Source: PLoS One. 2013 Dec 17;8(12):e82715. doi: 10.1371/journal.pone.0082715 (PMC3866189; doi:10.1371/journal.pone.0082715)
Supplement: Table S2 — The names and the corresponding abbreviations of the regions of interest (ROIs). (DOC) [file pone.0082715.s003.doc]

**Table S2.** The names and the corresponding abbreviations of the regions of interest (ROIs) defined in an AAL template image (45 regions for each hemisphere) described by Tzourio-Mazoyer et al. (2002).

| Regions | Abb. | Regions | Abb. |
| --- | --- | --- | --- |
| Precentral gyrus | PreCG | Lingual gyrus | LING |
| Superior frontal gyrus (dorsal) | SFGdor | Superior Occipital gyrus | SOG |
| Orbitofrontal cortex (superior) | ORBsup | Middle occipital gyrus | MOG |
| Middle frontal gyrus | MFG | Inferior occipital gyrus | IOG |
| Orbitofrontal cortex (middle) | ORBmid | Fusiform gyrus | FFG |
| Inferior frontal gyrus (opercular) | IFGoperc | Postcentral gyrus | PoCG |
| Inferior frontal gyrus (triangular) | IFGtriang | Superior parietal gyrus | SPG |
| Orbitofrontal cortex (inferior) | ORBinf | Inferior parietal lobule | IPL |
| Rolandic operculum | ROL | Supramarginal gyrus | SMG |
| Supplementary motor area | SMA | Angular gyrus | ANG |
| Olfactory | OLF | Precuneus | PCUN |
| Superior frontal gyrus (medial) | SFGmed | Paracentral lobule | PCL |
| Orbitofrontal cortex (medial) | ORBmed | Caudate | CAU |
| Rectus gyrus | REC | Putamen | PUT |
| Insula | INS | Pallidum | PAL |
| Anterior cingulate gyrus | ACG | Thalamus | THA |
| Middle cingulate gyrus | MCG | Heschl gyrus | HES |
| Posterior cingulate gyrus | PCG | Superior temporal gyrus | STG |
| Hippocampus | HIP | Temporal pole (superior) | TPOsup |
| Parahippocampal gyrus | PHG | Middle temporal gyrus | MTG |
| Amygdala | AMYG | Temporal pole (middle) | TPOmid |
| Calcarine cortex | CAL | Inferior temporal gyrus | ITG |
| Cuneus | CUN |  |  |
