# Supplementary material for: Disrupted Topological Organization in Whole-Brain Functional Networks of Heroin-Dependent Individuals: A Resting-State fMRI Study
Source: PLoS One. 2013 Dec 17;8(12):e82715. doi: 10.1371/journal.pone.0082715 (PMC3866189; doi:10.1371/journal.pone.0082715)
Supplement: Table S3 — The mathematical definitions and descriptions of global metrics and nodal metrics. (DOC) [file pone.0082715.s004.doc]

**Table S3.** The mathematical definitions and descriptions of global metrics and nodal metrics in the current study according to Rubinov et al. (2010).

| Network parameters | | Definitions | Descriptions |
| --- | --- | --- | --- |
|  | Clustering coefficient | $C_{p}=\frac{1}{N}\sum_{i\in G} \frac{E_{i}}{D_{nod}(i)(D_{nod}(i)-1)/2}$ | *D*_nod_(*i*) is the degree of node *i*, *E*_i_ is the number of edges in *G*_i_ (the subgraph of node *i*), *N* is the number of nodes in network. |
|  | Characteristic path length | $L_{p}=\frac{1}{1/(N(N-1))\sum_{j\neq i\in G} 1/L_{ij}}$ | *L*_ij_ is the shortest path length between nodes *i* and *j*. |
|  | Normalized clustering  coefficient | $\gamma=C_{p}^{real}/C_{p}^{rand}$ | *C*real p is the clustering coefficient of a real network, *C*rand p is the mean clustering coefficient of 100 matched random networks. |
| Global metrics | Normalized characteristic  path length | $\lambda=L_{p}^{real}/L_{p}^{rand}$ | *L*real p is the clustering coefficient of a real network, *L*rand p is the mean clustering coefficient of 100 matched random networks. |
|  | Small-worldness | $\sigma=\gamma/\lambda$ | See above |
|  | Global efficiency | $E_{glob}=\frac{1}{N(N-1)}\sum_{j\neq i\in G} 1/L_{ij}$ | *L*_ij_ is the shortest path length between nodes *i* and *j*. |
|  | Local efficiency | $E_{loc}=\frac{1}{N}\sum_{i\in G} E_{glob}(i)$ | *E*_glob_(*i*) is the global efficiency of *G*_i_. |
|  | Nodal degree | $D_{nod}(i)=\sum_{j\neq i} e_{ij}$ | *e*_ij_ is the (*i*, *j*)th element in the obtained binarized correlation matrix. |
| Nodal metrics | Nodal efficiency | $E_{nod}(i)=\frac{1}{N}\sum_{j\neq i} 1/L_{ij}$ | *L*_ij_ is the shortest path length between nodes *i* and *j*, *N* is the number of nodes in network. |
|  | Betweenness centrality | $N_{bc}(i)=\sum_{j\neq i\neq k} \frac{\delta_{jk}(i)}{\delta_{jk}}$ | *δ*j k is the number of shortest paths from node *j* to *k*, *δ*_jk_(*i*) is the number of shortest paths from node *j* to *k* that pass through node *i*. |
