# Supplementary material for: Disrupted Topological Organization in Whole-Brain Functional Networks of Heroin-Dependent Individuals: A Resting-State fMRI Study
Source: PLoS One. 2013 Dec 17;8(12):e82715. doi: 10.1371/journal.pone.0082715 (PMC3866189; doi:10.1371/journal.pone.0082715)
Supplement: Table S4 — Hub regions of the brain functional networks detected in the heroin-dependent individuals (HDIs) and normal controls (NCs). (DOC) [file pone.0082715.s005.doc]

**Table S4.** Hub regions of the brain functional networks detected in the heroin-dependent individuals (HDIs) and normal controls (NCs).

| HDIs | | | NCs | | |
| --- | --- | --- | --- | --- | --- |
| Regions | Classification | $N_{bc}^{norm}$ | Regions | Classification | $N_{bc}^{norm}$ |
| MFG.R | Association | 1.85 | PCUN.R | Association | 1.59 |
| **SMG.R** | Association | 1.84 | **SMG.R** | Association | 1.58 |
| **MTG.R** | Association | 1.83 | **MTG.R** | Association | 1.96 |
| **MCG.L** | Paralimbic | 1.81 | **MCG.L** | Paralimbic | 2.31 |
| **MCG.R** | Paralimbic | 1.76 | **MCG.R** | Paralimbic | 1.89 |
| **STG.R** | Association | 1.66 | **STG.R** | Association | 1.84 |
| **STG.L** | Association | 1.66 | **STG.L** | Association | 2.01 |
| **MOG.L** | Association | 1.62 | **MOG.L** | Association | 1.69 |
| **MTG.L** | Association | 1.59 | **MTG.L** | Association | 1.62 |
| **SFGdor.R** | Association | 1.58 | **SFGdor.R** | Association | 1.45 |
| PCUN.L | Association | 1.52 | MFG.L | Association | 1.67 |
| **IFGoperc.R** | Association | 1.50 | **IFGoperc.R** | Association | 1.48 |
| **SMA.R** | Association | 1.46 | **SMA.R** | Association | 1.65 |
| PoCG.L | Primary | 1.42 | TPOsup.R | Paralimbic | 1.54 |

The bold text indicates that these hub regions were identified in the functional networks of both of the groups. $N_{bc}^{norm}$: the normalized betweenness centrality.
