# Supplementary material for: Disrupted Topological Organization in Whole-Brain Functional Networks of Heroin-Dependent Individuals: A Resting-State fMRI Study
Source: PLoS One. 2013 Dec 17;8(12):e82715. doi: 10.1371/journal.pone.0082715 (PMC3866189; doi:10.1371/journal.pone.0082715)
Supplement: Table S5 — Decreased functional connections in the heroin-dependent individuals (HDIs) as compared to the normal controls (NCs). (DOC) [file pone.0082715.s006.doc]

**Table S5.** Decreased functional connections in the heroin-dependent individuals (HDIs) as compared to the normal controls (NCs).

| Region 1 | Category | Region 2 | Category | *t*-value | Inter-lobe  (Yes or No) |
| --- | --- | --- | --- | --- | --- |
| ORBmid.L | Frontal | ITG.L | Temporal | 3.351 | Y |
| MCG.L | Frontal | TPOsup.R | Temporal | 3.274 | Y |
| ORBmid.L | Frontal | THA.L | Subcortical | 3.200 | Y |
| PCUN.R | Parietal | MTG.R | Temporal | 3.138 | Y |
| ORBmid.L | Frontal | TPOmid.L | Temporal | 3.096 | Y |
| MFG.R | Frontal | THA.L | Subcortical | 2.983 | Y |
| PCG.L | Frontal | STG.L | Temporal | 2.828 | Y |
| ORBmid.L | Frontal | PHG.L | Subcortical | 2.801 | Y |
| IPL.L | Parietal | ITG.L | Temporal | 2.792 | Y |
| IFGtriang.L | Frontal | ITG.L | Temporal | 2.759 | Y |
| PCUN.L | Parietal | MTG.R | Temporal | 2.739 | Y |
| PCG.R | Frontal | STG.L | Temporal | 2.694 | Y |
| PCG.R | Frontal | MTG.R | Temporal | 2.683 | Y |
| PCG.R | Frontal | HES.R | Temporal | 2.612 | Y |
| IPL.L | Parietal | PCUN.R | Parietal | 2.568 | N |
| MFG.L | Frontal | ITG.L | Temporal | 2.425 | Y |
| MFG.R | Frontal | SOG.R | Occipital | 2.175 | Y |
| PHG.L | Subcortical | TPOsup.R | Temporal | 2.167 | Y |
| MCG.L | Frontal | PCG.R | Frontal | 1.956 | No |

Connections are listed in descending order of statistical significance (*p* < 0.05). Positive *t*-values mean NCs > HDIs. These connections formed a connected network, as determined by a network-based statistic method (*p* < 0.001, corrected). See Figure 4 for a graphical presentation of these connections. See Table S2 for the abbreviations of regions.
